# From threat to repair: metaphorical architecture and cognitive mobilization in Trumpist governing-coalition texts

**DOI:** 10.3389/fpsyg.2026.1888538

**Published:** 2026-08-03

**Authors:** Xuhai Chen, Zhiheng Zheng, Jiaxuan Lv

**Affiliations:** College of International Studies, National University of Defense Technology, Nanjing, Jiangsu, China

**Keywords:** BERTopic, cognitive mobilization, critical metaphor analysis, elite discourse, emotion appraisal, Trumpism

## Abstract

**Background:**

Research on Trumpism has concentrated on Donald Trump’s campaign speeches and social media, while the long-form writings of governing-coalition elites remain underexamined. Book-length texts offer more complete argumentative structures than tweets or short speeches, providing a fuller view of how elites construct crisis narratives through metaphor.

**Methods:**

Six book-length works published before their authors entered core positions in the Trump governing coalition were analyzed using an integrated framework combining BERTopic topic modeling, manual metaphor identification, source-target domain mapping, actor-positioning coding, and multi-label emotion appraisal within corpus-assisted critical metaphor analysis.

**Results:**

Across the six texts, 2,074 contextually verified linguistic metaphors were identified, concentrated in six focal source domains—war/conflict, decline/fall, corruption/disease, invasion/infiltration, betrayal/abandonment, and revival/reconstruction—forming a pronounced crisis–repair asymmetry: threat-oriented domains account for over 90% of instances, while revival/reconstruction provides a limited but emotionally concentrated repair script. Actor positioning reveals “us vs. internal them” as the predominant pattern, locating antagonists inside the political community. Emotion-appraisal profiles differentiate these domains: anger and pride/dignity characterize war/conflict, loss/nostalgia marks decline/fall, disgust and humiliation cluster in corruption/disease, and hope and sacredness define revival/reconstruction.

**Conclusion:**

These findings indicate a stable, cross-issue crisis-and-repair metaphorical architecture that extends beyond a single leader’s rhetoric, functioning as a shared cognitive mobilization structure at the textual level. The study contributes theoretically by showing how differentiated metaphor-emotion profiles organize crisis construction, blame attribution, and repair imagination in political texts. It also contributes methodologically by offering an operational pathway for integrating computational topic modeling with corpus-assisted critical metaphor analysis.

## 1 Introduction

A central concern in discourse analysis, argumentation studies, and political communication has long been how political discourse constructs reality, shapes cognition, and guides visions of political action through linguistic strategies (Fairclough, 2009). Contemporary right-wing populism continues to expand. Political elites often employ compressed narrative frames that transform complex socio-economic contradictions into crisis scenarios—scenarios that are easier to perceive and more strongly charged with moral evaluation (Atmawijaya, 2025). Metaphor is a cognitive mechanism that structures political experience, demarcates value positions, channels responsibility attribution, and activates imagined courses of action (Lakoff and Johnson, 2003). In this light, metaphorical choices in political texts serve as an entry point for understanding how political actors construct the world, interpret crises, and imagine possible solutions.

Trumpism combines right-wing populism, nativism, and anti-establishment demands into a narrative formation that extends beyond a single leader’s rhetoric—making it a limiting case for the analysis. It has reshaped the landscape of domestic political competition in the United States and has influenced U.S. foreign strategy, narratives of national identity, and modes of social mobilization (Löfflmann, 2022). At the discursive level, Trumpism relies on crisis narratives: the nation is described as being in decline, institutions as corroded, borders as under threat, the people as betrayed by elites, and political action as endowed with the mission of reviving, taking back, and rebuilding the country (Homolar and Scholz, 2019). These narrative elements cast issues such as immigration, education, public health, judicial institutions, economic order, national security, and global competition into a shared cognitive frame of “America in crisis,” thereby giving otherwise dispersed policy disputes a common moral structure and emotional orientation.

Existing studies of Trumpism have examined leader style (Caputo and Aragusuku, 2024), voter composition (Bhambra, 2017; Lamont et al., 2017; Monnat and Brown, 2017; Mutz, 2018), identity politics (Bobo, 2017; Armaly et al., 2022), right-wing populist thought (Bonikowski, 2017), and social media communication. These studies have revealed the social foundations and communicative mechanisms behind the rise of Trumpism. Nevertheless, two limitations remain in the existing literature. Research has been concentrated on Donald Trump’s campaign speeches, social media discourse, and political performances. Insufficient attention has been paid to the long-form, systematic writings of the political elites surrounding him. Compared with tweets, campaign slogans, or short speeches, book-length works offer more complete argumentative structures, more stable value frameworks, and clearer expressions of worldview. This makes them especially suitable for examining the cognitive schemas that political elites had already formed before entering senior government positions. Existing scholarship has devoted considerable attention to the social foundations, communicative style, and emotional mobilization effects of Trumpism. Less systematic attention has been paid to how its texts internally construct political reality through metaphorical resources and connect crisis perception with imagined courses of action. Trumpism needs to be explained as both a socio-political phenomenon and a political cognitive system with its own internal organizing capacity.

This study uses six book-length works, published before their authors later entered core positions in the Trump governing coalition, as its corpus. These texts differ in genre, theme, and authorial background, covering such issues as white working-class experience, economic nationalism, anti-establishment politics, culture war, deep-state narratives, and public health controversies. This heterogeneity makes them an appropriate set of texts for testing the capacity of Trumpist narrative to integrate diverse issues. If these heterogeneous texts repeatedly draw on similar metaphorical resources and patterns of emotional evaluation across different issue domains, this would indicate that Trumpist elite discourse may contain a shared cognitive framework that transcends individual style. This study does not examine whether the authors engaged in any actual collaborative writing. Instead, it focuses on how their texts reproduce similar patterns of crisis construction, responsibility attribution, and revival imagination within their respective issue areas.

The pre-office timing of these texts is methodologically significant: it ensures that the metaphorical patterns examined below are not shaped by the institutional discourse constraints of executive-branch positions, though it does not—and cannot—provide direct access to the authors’ private cognitive states. The analysis therefore reconstructs textual-level conceptual patterns rather than making claims about individual psychology.

Three questions organize the analysis:

*RQ1*: What recurrent metaphorical source domains emerge from the pre-office writings of political figures who later entered core positions in the Trump governing coalition, and how are these source domains distributed across authors and issue contexts?

*RQ2*: How do the retained source domains construct crisis perception, position collective actors as an in-group (“us”) and as external or internal out-groups (“them”), assign responsibility, and legitimize imagined courses of action across different target domains?

*RQ3*: How do emotion-appraisal profiles differentiate the six metaphorical source domains, and how do these evaluative contrasts help connect crisis construction, blame attribution, and repair-oriented cognitive mobilization?

To address these questions, this study adopts a corpus-assisted critical metaphor analysis. First, BERTopic topic modeling is used to identify the core issue contexts of the six texts, thereby providing macro-level semantic anchors for metaphor analysis. Second, candidate metaphorical expressions are identified through keyword retrieval, contextual co-occurrence analysis, and manual verification, and recurrent source-domain families are then consolidated for focused analysis. Third, metaphor-bearing paragraph–source-domain pairings are coded for in-group/out-group actor positioning. Fourth, multi-label emotion appraisal is used to compare diagnostic evaluative profiles across the six source domains and to examine how these profiles contribute to the textual shift from crisis construction to repair-oriented cognitive mobilization.

The aim of this study is to reveal how Trumpist elite texts use a relatively stable set of metaphorical resources to frame policy disputes across different fields as crisis narratives with a shared direction. It further explains how this metaphorical system connects cognitive frames, emotional evaluations, and imagined courses of action at the textual level. In doing so, this study seeks to shift the analytical focus of Trumpism research from the discourse of a single leader to the collective narrative structure of governing-coalition elites. It also provides an operational research pathway for integrating computational methods with critical metaphor analysis.

## 2 Theoretical framework

The analytical framework integrates three complementary bodies of work. Conceptual metaphor theory supplies the core distinction between source and target domains and explains how experiential structures shape political cognition. Critical metaphor analysis adds what purely cognitive accounts underplay: metaphor’s role in power relations, identity demarcation, and ideological production. Political emotion theory addresses a gap in both traditions—specifying how metaphor carries differentiated emotional appraisals that connect crisis perception to imagined courses of action.

### 2.1 Conceptual metaphor as a structure of political cognition

Lakoff and Johnson treat metaphor not as rhetorical ornament but as a cognitive mechanism for structuring abstract experience through source-target mappings. People often use concrete, familiar, and perceptible source domains to understand abstract and complex target domains, structuring experience through systematic mappings from source domains to target domains (Lakoff and Johnson, 2003). The analytical focus of metaphor, therefore, should be on how one experiential structure is projected onto another category of social objects, rather than on whether individual expressions are vivid. This view also explains why metaphor can function as a persuasive frame: it does not only name an abstract object, but also directs attention to particular causal relations, moral evaluations, and possible responses. Experimental evidence shows that even subtle metaphorical cues can influence how people reason about social problems and what solutions they prefer (Thibodeau and Boroditsky, 2011). A meta-analysis of metaphorical framing in political discourse further confirms that both word-level and concept-level metaphorical frames can affect political beliefs and attitudes, with concept-level frames showing stronger effects (Brugman et al., 2019).

Political discourse depends on this mechanism because target domains such as state authority or institutional legitimacy admit multiple causal interpretations; metaphor selects among them. In political rhetoric, metaphor therefore links cognition with persuasion and legitimation, because repeated source domains can help political actors create coherent belief systems and make particular courses of action appear reasonable or necessary (Charteris-Black, 2011). By invoking familiar experiences such as “war,” “disease,” “decline,” and “invasion,” metaphor lends complex political relations clearer perceptual contours (Semino et al., 2016). For example, the “war” metaphor usually maps political disagreement onto friend-enemy conflict, policy disputes onto battlefields, and political action onto attack, defense, or counterattack, thereby presupposing inferential paths such as victory and defeat, sacrifice, and mobilization (Flusberg et al., 2018). The “disease” metaphor understands institutional problems as bodily lesions and reform as diagnosis, removal of pathogens, and restoration of health (Musolff, 2010). The “decline” metaphor, through schemas such as falling, decay, and damage, structures social change as a historical trajectory from wholeness to ruin and from a higher position to a lower one (Keck, 2023).

Conceptual metaphor theory provides two analytical tools for this study. First, it explains how recurrent source-domain scripts can project experiential structures such as confrontation, boundary defense, pathology, descent, moral breach, and repair onto political target domains. Second, it enables the analysis to trace how these scripts shape political judgment in specific contexts, not merely by increasing the frequency of certain words but by organizing causal interpretation, moral evaluation, actor positioning, and imagined responses. The metaphor analysis that follows therefore treats source-domain frequency as only one part of the evidence; equal attention is given to how recurrent metaphorical expressions structure political reasoning within their surrounding argumentative contexts.

Conceptual metaphors and linguistic metaphorical expressions are treated as analytically distinct but empirically connected. Conceptual metaphors refer to source-target mappings that structure political cognition, whereas linguistic metaphorical expressions are the observable textual forms through which such mappings are realized in discourse. Since conceptual mappings cannot be directly observed in the corpus, this study uses recurrent and contextually verified linguistic expressions as evidence for reconstructing broader conceptual patterns.

### 2.2 Critical metaphor analysis: metaphor in power practices and ideological production

Conceptual metaphor theory explains the cognitive mechanism of metaphor. Critical metaphor analysis extends this approach by examining how metaphor functions within social power relations. Charteris-Black’s critical metaphor analysis integrates corpus methods, cognitive linguistics, and critical discourse analysis, emphasizing that metaphor performs persuasive, legitimizing, and ideological functions in political communication (Charteris-Black, 2004). It can be understood as involving three connected stages: metaphor identification, metaphor interpretation, and metaphor explanation. The present study follows this logic in a corpus-assisted form: metaphorically used expressions are first identified and contextually verified in section 3.3; their source-target mappings are then interpreted in section 5.1; and their ideological functions are explained in the subsequent critical discussion of crisis construction, responsibility attribution, boundary-making, and imagined political action.

Political metaphor studies have established broad source-domain classifications for examining how metaphor organizes political meaning and rhetorical force. In this tradition, metaphor is treated as a way of structuring political and world-political meanings, while source-domain categories provide a basis for identifying, counting, and comparing metaphorical patterns across political texts (Beer and Landtsheer, 2004; De Landtsheer, 2009, 2015). These studies offer a useful methodological background for the present analysis, which combines corpus-assisted metaphor identification with a focused examination of recurrent source-domain families in Trumpist elite texts. In the present corpus, the most recurrent and politically salient source-domain families are war/conflict, invasion/infiltration, corruption/disease, decline/fall, betrayal/abandonment, and revival/reconstruction. These domains organize political issues into scripts of crisis perception, responsibility attribution, boundary defense, and repair. The focus on these six retained focal domains allows the study to explain how metaphorical patterns connect issue contexts, emotional appraisal, and imagined courses of political action across heterogeneous elite texts.

In this light, metaphor helps people understand political reality and, at the same time, participates in constructing which objects should be regarded as threats, which actors should be held responsible, and which actions should be considered legitimate. Existing studies on Trump-related metaphors further show that metaphor is central to the framing and persuasive force of Trump’s political discourse. Trump’s speeches rely on conventional conceptual metaphors to frame issues such as immigration and the economy, while also constructing his political persona as a repairman, builder, healer, and warrior (Pilyarchuk and Onysko, 2018). Trump’s “wall” and “presidency as business” metaphors also provide access to the symbolic structure of his campaign and presidency, linking national vulnerability, exclusionary boundary-making, and the image of Trump as a protective dealmaker (Johnson and Stuckey, 2022). In the 2024 nomination acceptance speech delivered by Donald Trump, metaphor scenarios such as IMMIGRATION AS INVASION and NATION AS CONSTRUCTION PROJECT simplify complex issues, evoke emotional responses, and connect personal crisis with national renewal (Atmawijaya, 2025).

The function of political metaphor is selective. When a source domain is repeatedly invoked, it highlights certain aspects of a political object while suppressing other possible interpretive paths (Musolff, 2016). For example, describing immigration as an “invasion” or a “flood” foregrounds its scale, speed, and impact on communal boundaries, but tends to weaken the understanding of immigrants as workers, family members, or rights-bearing subjects (Santa Ana, 2021). Describing institutional problems as “corruption” or “cancer” strengthens the logic of internal pollution, lesion diffusion, and surgical purification, making gradual repair, procedural negotiation, and institutional compromise appear weak or insufficient (Abu Rumman et al., 2024). Describing political competition as a whole as “war” transforms opponents from legitimate competitors into enemies who must be defeated, thereby enhancing the legitimacy of conflictual expression and hardline action (Kalmoe, 2014).

These metaphorical frames should not be understood only as features of Trump’s personal style. Recent studies suggest that the Make America Great Again movement (MAGA) has developed as a status-based movement organized around perceived lost honor, declining esteem, institutional disrespect, anger at elites, and the desire to restore social recognition (Koenig and Mendelberg, 2026). MAGA discourse also circulates through digitally networked publics that connect grassroots actors, media hubs, political organizations, and elected officials, thereby extending Trumpist frames beyond a single leader’s rhetoric (Bennett and Livingston, 2025).

Critical metaphor analysis therefore requires researchers to examine both the cognitive structures and the ideological consequences of metaphor. Building on these studies, the present article shifts attention from Trump’s own speeches and campaign performances to the long-form writings of political elites who later entered the Trump governing coalition. Specifically, in relation to Trumpist elite texts, this study focuses on the functions of the retained focal metaphor types at four levels. How do they name threats and cast social problems as crises? How do they draw boundaries and divide the political community into “us” and “them”? How do they accomplish responsibility attribution by transforming complex structural problems into identifiable enemies, lesions, or betrayers? How do they generate action directives and provide a meaningful basis for political actions such as defense, cleansing, counterattack, taking back, and reconstruction?

### 2.3 Political emotion, appraisal, and cognitive mobilization

The political effects of metaphor derive from cognitive framing and from the emotional appraisals that metaphor carries. In appraisal approaches to emotion, emotions are understood as patterned evaluations of events, responsibility, threat, loss, agency, and possible response, rather than as undifferentiated affective intensity (Smith and Ellsworth, 1985). Research on political emotions shows that emotions such as anger, fear, loss, humiliation, betrayal, hope, and collective efficacy can influence political identification, responsibility attribution, and action intention (Neuman, 2007). Emotions are important in populist political communication, because populism often transforms political judgment into intense moral experience through oppositional structures such as “the people versus the elites,” “the community versus external threats,” and “past glory versus present decline” (Bliuc et al., 2026).

Different emotions perform different evaluative functions in political texts. Anger tends to be directed toward blameworthy objects and can transform social problems into accusations against specific groups or institutions (Valentino et al., 2011). Fear and anxiety are organized around potential threats; when external groups are described as security risks, cultural risks, or bodily risks, defensive policies are more likely to gain emotional support (Brader et al., 2008). Loss and nostalgia introduce vertical historical comparison and, through the narrative structure of “once great, now in decline,” provide an emotional precondition for revival discourse (Smeekes et al., 2021). Betrayal shifts crisis attribution inward, elevating political disagreement into a moral breach committed by elites against “the true people” (Ege and Springer, 2023). Hope and collective efficacy supply a mechanism of emotional transformation, giving crisis, anger, and loss a future-oriented outlet for action (Cohen-Chen and Van Zomeren, 2018).

Prior research suggests that source-domain scripts may make certain evaluative orientations more salient in political discourse. War/conflict and invasion/infiltration can foreground confrontation and boundary threat (Doquin De Saint Preux and Masid Blanco, 2021; Zawadzka-Paluektau, 2023); corruption/disease and decline/fall can support appraisals of contamination and loss (Van Der Velden et al., 2025; Valtorta et al., 2026). Betrayal/abandonment and revival/reconstruction can organize moral breach and repair-oriented agency, respectively (Hunger, 2024; Wojczewski, 2025). These relationships, however, should not be assumed to be fixed or one-to-one. The present study therefore uses multi-label emotion appraisal to compare which emotions are relatively over- or underrepresented across the six source domains and to identify the evaluative contrasts through which the texts organize crisis, blame, and repair.

This study treats metaphor–emotion relations as a textual analytical link through which metaphorical source domains acquire evaluative direction and support imagined action scripts. Here, cognitive mobilization refers to a textual process of meaning construction: how political texts, through metaphorical frames and emotional appraisal, enable readers to understand what counts as crisis, who should be held responsible, what kinds of action are legitimate, and how the future should be imagined. In this study, action directives are understood as textually inferable action scripts rather than direct evidence of audience behavior. Metaphors generate such directives by defining political objects as particular kinds of problems, assigning roles to relevant actors, and making certain responses appear appropriate, necessary, or morally justified. The study therefore analyzes a political cognitive chain with mobilizing potential, rather than making causal claims about actual audience behavior.

The following sections examine the distributional structure, issue embedding, and diagnostic emotion-appraisal profiles of the six retained focal source domains in the six pre-office book-length works.

## 3 Materials and methods

### 3.1 Corpus construction

This study uses six book-length works, published before their authors later entered core positions in the Trump governing coalition, as its corpus (Table 1). This selection is based on two considerations. Compared with post-office policy documents, media interviews, or social media texts, book-length works contain more complete argumentative structures and more stable value frameworks. They are therefore more suitable for examining the political cognitive schemas that these authors had already formed before assuming senior government positions (Olson et al., 2012).

**TABLE 1:** Texts selected for this study.

Author	Position at time of publication	Subsequent position	Text	Year	Word count
J. D. Vance	Lawyer/venture capitalist	Vice President	*Hillbilly Elegy*	2016	79,782
Marco Rubio	Senator from Florida	Secretary of State	*Decades of Decadence*	2023	79,863
Tulsi Gabbard	Former Representative	Director of National Intelligence	*For Love of Country*	2024	69,780
Pete Hegseth	Host / writer	Secretary of Defense	*Battle for the American Mind*	2022	79,234
Kash Patel	Former Chief of Staff to the Acting Secretary of Defense	Director of the Federal Bureau of Investigation	*Government Gangsters*	2023	74,171
Robert F. Kennedy Jr.	Chairman and Chief Litigation Counsel, Children’s Health Defense	Secretary of Health and Human Services	*The Real Anthony Fauci*	2021	190,132

Second, these texts were selected because they represent highly visible and widely circulated pre-office publications by each author. Their prominence is supported by multiple indicators, including national bestseller status, publisher-reported sales, Amazon category rankings, and substantial reader-review counts. Several titles, including *Hillbilly Elegy*, *Battle for the American Mind*, *For Love of Country*, *Government Gangsters*, and *The Real Anthony Fauci*, appeared on major bestseller lists or were marketed as national bestsellers, while *Decades of Decadence* showed category-level visibility on Amazon. This criterion was applied before metaphor coding and source-domain analysis, and it therefore reduces the risk of a direct sampling circularity in which texts would be selected because they already contained conflict metaphors or crisis narratives.

At the same time, public prominence should not be treated as entirely independent of conflictual narration. In political elite writing, and especially in populist political nonfiction, widely circulated books may gain visibility partly because they dramatize public problems through crisis, moral opposition, or antagonistic storytelling (Kirch, 2018; Spratford, 2026). The present study therefore does not claim that the corpus is free from the conflictual tendencies of its genre. Rather, it treats these tendencies as part of the discourse field under investigation. The six authors differ in professional background, issue domain, and textual genre, covering such areas as class experience, economic nationalism, anti-establishment politics, culture war, national security, and public health controversies. This heterogeneity makes the corpus suitable for testing whether elite discourse within the Trump governing coalition repeatedly draws on similar metaphorical resources and cognitive frames across different issue domains.

For corpus processing, the six EPUB-format books were parsed using Python. The main text was extracted, and paratextual materials—tables of contents, copyright information, notes, and references—were removed. A dedicated stop-word list and a proper-noun whitelist for English political texts were then constructed. After cleaning, normalization, and noise reduction, the texts were segmented into paragraphs, resulting in a paragraph-level corpus. This study uses the paragraph as the basic unit of analysis because paragraphs tend to carry relatively complete propositional networks. They preserve the semantic coherence between metaphorical expressions and their surrounding context, while also allowing the analytical granularity to align with topic modeling and emotion appraisal. Sentence-level analysis may fragment the argumentative function of metaphors, whereas document-level analysis is too coarse to capture the specific correspondences among metaphor, issue context, and emotional evaluation.

### 3.2 Topic modeling and issue localization

This study uses topic modeling as a tool for macro-level issue localization rather than as a direct source of conclusions for metaphor interpretation. Because the meaning of metaphor depends on the issue context in which it appears, the major issue structures need to be identified across the six texts before proceeding to metaphor identification and critical close reading. This provides semantic anchors for the subsequent analysis.

BERTopic was used to conduct semantic clustering on the paragraph-level corpus (Mendonça and Figueira, 2024). Specifically, the Sentence-Transformers pretrained model all-mpnet-base-v2 was used to encode paragraphs into semantic vectors. UMAP was then applied for dimensionality reduction with the following parameters: n_neighbors = 60, n_components = 5, and min_dist = 0.0. HDBSCAN was used for density-adaptive clustering with min_cluster_size = 60 and min_samples = 15. Finally, c-TF-IDF was used to extract the top 12 representative keywords for each cluster. To improve the robustness of the results, models were trained under multiple parameter combinations, and the results were manually evaluated by considering topic coherence, keyword interpretability, and the quality of representative paragraphs. Paragraphs with highly dispersed semantics or topics that could not be interpreted stably were assigned to outlier topics and were not included in the main issue aggregation.

The BERTopic results serve three main purposes. First, they identify the core issue contexts across the six texts. Second, they allow comparison of issue emphasis among different authors. Third, they provide representative paragraph samples for subsequent metaphor identification and critical close reading. Notably, topic distribution reflects the macro-level issue structure of the corpus and cannot be directly equated with the degree of sharedness across authors. The judgment concerning shared metaphorical frames in the following sections is based on the manual verification of metaphorical instances, the distribution of source domains, the strength of association across authors, and close reading of specific textual evidence.

In the process of macro-level issue aggregation, this study follows the principle of “dominant semantics first.” For topics involving multiple issue areas, the topic is assigned to one major category according to its keywords, representative paragraphs, and primary argumentative object. Its secondary semantic features may be discussed in qualitative interpretation, but they are not repeatedly counted in statistical tests, so as to avoid category overlap in contingency tables.

### 3.3 Metaphor identification, actor positioning, and emotion appraisal

Within the issue framework anchored by topic modeling, this study identifies linguistic metaphorical expressions as observable textual units and interprets them as evidence for broader conceptual source-target mappings. For the purposes of this study, a linguistic expression is identified as metaphorical when its contextual meaning differs from its more basic, concrete, or experientially immediate meaning, and when the contextual meaning can be understood through comparison with that basic meaning (Steen et al., 2010). “Basic meaning” refers to the more concrete, bodily, or directly experienced sense of a lexical unit, whereas “contextual meaning” refers to the meaning that the unit conveys in the specific passage under analysis. Thus, an expression is not coded as metaphorical merely because it belongs to a lexical field commonly associated with metaphor, such as war, disease, decline, or borders. It is coded as metaphorical only when the context activates a cross-domain mapping through which an abstract political object, relation, or process is structured in terms of a more concrete experiential domain.

Conceptual metaphor theory and previous studies of political metaphor were used as sensitizing resources for recognizing possible source-domain patterns, but they did not provide a fixed typology to be imposed on the corpus. Candidate metaphorical expressions were first collected through keyword-in-context searches, BERTopic representative paragraphs, and close reading across authors and issue contexts. At this exploratory stage, source-domain labels were assigned in an open-ended manner. In addition to the domains eventually retained for focused analysis, broader or auxiliary domains such as everyday life, nature, technical/mechanical process, body, market, journey, vision/light, family, religion, game/sport, and fortress/defense were also considered whenever such mappings appeared in the corpus.

The initial open-ended coding identified more than twenty candidate source-domain labels. These labels were then consolidated through an iterative comparison of contextual meaning, basic meaning, source-domain coherence, and recurrence across authors or issue contexts. Conceptually adjacent labels were merged when they projected similar inferential scripts onto political target domains. For example, expressions associated with combat, defense, siege, and weaponization were consolidated under war/conflict, while expressions associated with boundary breach, inflow, encroachment, and infiltration were consolidated under invasion/infiltration. Other labels were not retained for focused analysis when they appeared only locally, lacked a stable source-target mapping across the corpus, or functioned mainly as auxiliary imagery rather than as a recurrent frame for defining political problems, positioning collective actors, or implying courses of action.

On this basis, six focal source domains were retained for systematic analysis: war/conflict, invasion/infiltration, corruption/disease, decline/fall, betrayal/abandonment, and revival/reconstruction. These domains should therefore be understood as corpus-derived analytical categories, not as a predetermined crisis-oriented typology. The crisis-repair pattern discussed in the Results section emerges from their distributional dominance, cross-contextual recurrence, actor-positioning patterns, and emotion-appraisal profiles, rather than from the initial coding design.

Around the retained focal source domains, keyword retrieval lists were constructed to locate candidate metaphorical expressions. To make the selection process transparent, the lists were developed through a three-step procedure. First, seed terms were selected according to the source-domain scripts identified in conceptual metaphor theory and previous studies of political metaphor: confrontation, attack, defense, and victory/defeat for war/conflict; boundary violation and uncontrolled entry for invasion/infiltration; pathology, decay, and contamination for corruption/disease; downward movement and collapse for decline/fall; broken trust and moral breach for betrayal/abandonment; and repair, restoration, and renewed agency for revival/reconstruction. Second, these seed terms were expanded through BERTopic representative paragraphs, keyword-in-context inspection, and close reading across authors and issue contexts. Third, lexical variants, inflectional forms, and recurrent collocational expressions were retained when they were conceptually relevant to the same source-domain script and recurrent or interpretively salient in the corpus. Keyword retrieval was therefore used only to generate candidate instances and was not treated as an automatic criterion for identifying metaphor. Each candidate expression was manually checked in sentence, local-window, and paragraph context by comparing its contextual meaning with its more basic meaning. Only when the contextual meaning differed from the basic meaning and could be understood through a cross-domain mapping was the expression confirmed as a linguistic metaphorical expression. Source-domain assignment was made only after this metaphorical judgment had been made.

To make the identification procedure more transparent, Table 2 provides three worked examples drawn from the coded corpus.

**TABLE 2:** Worked examples of metaphor identification.

Candidate expression	Textual context	Contextual meaning	Basic meaning	Judgment
“Rotten at the top”	Patel describes the federal bureaucracy as “rotten at the top.”	Institutional corruption, dysfunction, and internal decay within federal agencies.	Physical decay or decomposition of organic matter.	Metaphorical. Institutional dysfunction is understood through organic decay; assigned to the corruption/disease source domain.
“Total war against President-elect Trump”	Patel describes political and institutional opposition as a “total war.”	A sustained political and institutional struggle.	Armed conflict between nations, armies, or organized military groups.	Metaphorical. Non-military political struggle is understood through military confrontation; assigned to the war/conflict source domain.
“Civil war”	Patel refers to the suffering of the Syrian people during their “civil war.”	Actual armed conflict in Syria.	Armed conflict between organized political or military groups.	Literal use. The contextual meaning does not differ substantially from the basic meaning, so the expression is excluded from metaphor counts.

These examples show that metaphor identification in this study depends on contextual reading rather than keyword occurrence alone. The same lexical item may be metaphorical in one context and literal in another. For instance, “war” is counted as metaphorical when it structures political, institutional, cultural, or public-health disputes as military confrontation, but it is excluded when it refers to actual armed conflict. In this way, the study distinguishes among keyword occurrence, literal use, and linguistic metaphorical expression, so as to avoid equating word frequency directly with metaphor analysis.

This distinction is methodological rather than substantive. It does not deny that many of the texts are organized around politically conflictual objects, such as culture-war education debates, public-health controversies, deep-state narratives, border politics, and global security issues. Nor does it claim that conflict metaphors are incidental to the corpus. Instead, the procedure separates three levels that could otherwise be conflated: the presence of conflict-related topics, the occurrence of conflict-related lexical items, and the contextual judgment that an abstract political or institutional target is being structured through a concrete source-domain script of confrontation, attack, defense, victory, or defeat. War/conflict expressions were therefore counted only when they involved such a source-target mapping. In this sense, the high frequency of conflict metaphors is treated as an empirical finding of the analysis, not as a premise of the research design or as the automatic result of raw keyword retrieval.

Building on this contextual identification procedure, Table 3 summarizes the keyword retrieval lists, source-domain scripts, inclusion rationales, and main exclusion rules used to generate candidate instances for manual verification.

**TABLE 3:** Summary of keyword retrieval patterns and contextual verification rules^1^.

Source domain	Source-domain script and inclusion rationale	Normalized keyword examples from retrieval patterns	Main exclusion rule
War/conflict	Terms were included when they potentially activated a script of confrontation, attack, defense, mobilization, and victory or defeat.	War, warfare, battle, battlefield, battlefront, fight, fight back, fighter, enemy, defend, attack, combat, frontline, weapon, weaponize, siege, victory, defeat, ammunition, arsenal, warrior, surrender, capitulate	Literal references to actual armed conflict, military operations, soldiers, battlefields, or war experience were excluded unless an abstract political target was structured through the war/conflict domain.
Invasion/infiltration	Terms were included when they potentially construed political, cultural, institutional, or demographic processes as boundary violation, uncontrolled entry, or external pressure.	Invasion, invade, invader, flood, surge, wave, swarm, pour, infiltrate, infiltration, infiltrator, overrun, breach, tide, influx, encroach, encroachment, penetrate, penetration	Literal physical entry, natural flooding, military invasion, or concrete movement was excluded unless used to map an abstract political process onto boundary violation.
Corruption/disease	Terms were included when they potentially mapped institutional, political, cultural, or moral problems onto pathology, decay, infection, toxicity, pollution, or cleansing.	Corrupt, corruption, rot, rotten, decay, disease, cancer, cancerous, virus, infect, infection, infectious, poison, poisonous, toxic, toxicity, sick, sickness, plague, epidemic, cure, cleanse, purge, purify, purification, contaminate, contamination	Literal medical, epidemiological, biological, or public-health references were excluded unless they structured an abstract political or institutional target.
Decline/fall	Terms were included when they potentially constructed national, institutional, cultural, or economic change as downward movement, collapse, deterioration, damage, or loss.	Decline, fall, fallen, collapse, crumble, ruin, ruined, sink, sank, sunk, deteriorate, deterioration, fade, dying, broken, lost, downfall, crisis	Literal physical falling, actual death, physical breakage, or ordinary loss was excluded unless an abstract social or political target was mapped onto decline or collapse.
Betrayal/abandonment	Terms were included when they potentially construed political crisis as broken trust, moral breach, elite betrayal, or abandonment of the people.	Betray, betrayal, treason, treasonous, stabbed in the back, sold out, sell out, abandon, abandonment, forsake, forsaken, lied to, cheat, deceive, traitor, turn against	Purely interpersonal betrayal or private relationship contexts were excluded unless mapped onto a political community, institution, or collective subject.
Revival/reconstruction	Terms were included when they potentially constructed political action as repair, rebuilding, restoration, recovery, reclaiming, or collective renewal after crisis.	Restore, restoration, revive, revival, rebuild, rebuilt, reclaim, take back, taking back, took back, renew, renewal, rebirth, resurgence, save, rescue, fix, heal, rise again, rising again, make America great again, great again	Literal physical repair, literal rescue, or medical healing was excluded unless used to structure an abstract political, national, institutional, or cultural target.

^1^The keyword examples in this table are normalized from the regular-expression retrieval patterns used in the candidate extraction script. They were used only to retrieve candidate expressions and were not treated as automatic evidence of metaphor. Each keyword hit was checked in sentence, local-window, and paragraph context. A candidate was retained only when its contextual meaning differed from its more basic meaning and could be interpreted through a cross-domain mapping. Literal uses and contextually irrelevant hits were excluded from the final metaphor counts.

After metaphor identification, the analytical focus shifts to source-target domain mappings. This study examines how the six retained focal source domains are projected onto such target domains as U.S. political institutions, immigration and borders, the education system, public health, economic order, elite groups, and global competition. It further analyzes how these mappings define the nature of political objects, assign value evaluations to them, and embed them in broader ideological narratives. Therefore, the focus of this study is not simply to count the frequency of metaphor keywords, but to explain how metaphors structure crisis perception, responsibility attribution, and imagined courses of action in specific passages.

To examine how metaphorical boundary-making organizes collective actors, this study uses an actor-positioning coding layer within the metaphor analysis. The coding unit is the metaphor-bearing paragraph–source-domain pairing rather than the individual keyword occurrence, because a single paragraph may contain multiple metaphorical expressions from the same source domain. This procedure yielded 1,704 paragraph–source-domain pairings from the 2,074 confirmed metaphorical occurrences. Each unit was coded for positioning configuration, in-group label and role, out-group location and role, action script, and explicitness. Action script was coded as the contextually inferable response logic projected by a metaphor-bearing paragraph–source-domain pairing, rather than as an explicit behavioral instruction. Its identification was based on the interaction among the inferential script of the source domain, the roles assigned to in-group and out-group actors, and, where relevant, the paragraph-level emotional appraisal that made certain responses appear appropriate, necessary, or morally justified. The coding did not rely only on pronouns such as “we,” “us,” “they,” and “them,” but also included collective nouns and politically salient group labels when they functioned as discourse-internal equivalents of in-groups or out-groups. Abstract references without an identifiable collective actor were coded as having no collective positioning.

To examine the evaluative differentiation of the six source domains, the study applied a 12-category multi-label emotion-appraisal scheme at the paragraph level, including anger, fear/anxiety, loss/nostalgia, humiliation, betrayal, justice appeal, hope, pride/dignity, sacredness, determination, collective efficacy, and disgust/pollution. Emotion labels were aligned with the 1,704 paragraph-source-domain pairings and compared across source domains using chi-square tests and ASR. The analysis focuses on emotions that are significantly over- or underrepresented in particular source domains, rather than treating frequently occurring emotions as domain-specific findings. Emotion appraisal is used as an interpretive layer for specifying the evaluative direction of metaphorical framing; it is not treated as a direct measure of authors’ intentions, audience emotions, or behavioral effects.

### 3.4 Analytical procedure and intercoder reliability

In sum, the analytical procedure of this study consists of five steps. First, a paragraph-level corpus is constructed and the texts are cleaned. Second, BERTopic is used to identify core issue contexts, thereby providing macro-level localization for metaphor analysis. Third, candidate metaphorical expressions are retrieved, manually verified, and then consolidated into six focal source-domain families for source-target mapping analysis. Fourth, actor-positioning coding is applied to metaphor-bearing paragraph–source-domain pairings in order to examine how the retained source domains position collective actors as an in-group (“us”) and as external or internal out-groups (“them”). Fifth, metaphor distributions and actor-positioning patterns are compared with multi-label emotion-appraisal profiles to identify evaluative contrasts among the six source domains, which are then interpreted through critical close reading. Through this procedure, the study combines quantitative distribution, actor-positioning analysis, emotion labels, and textual close reading to explain how the six retained focal metaphor types structure crisis perception, channel responsibility attribution, construct group boundaries, and activate imagined courses of action across different issue contexts.

**FIGURE 1:**
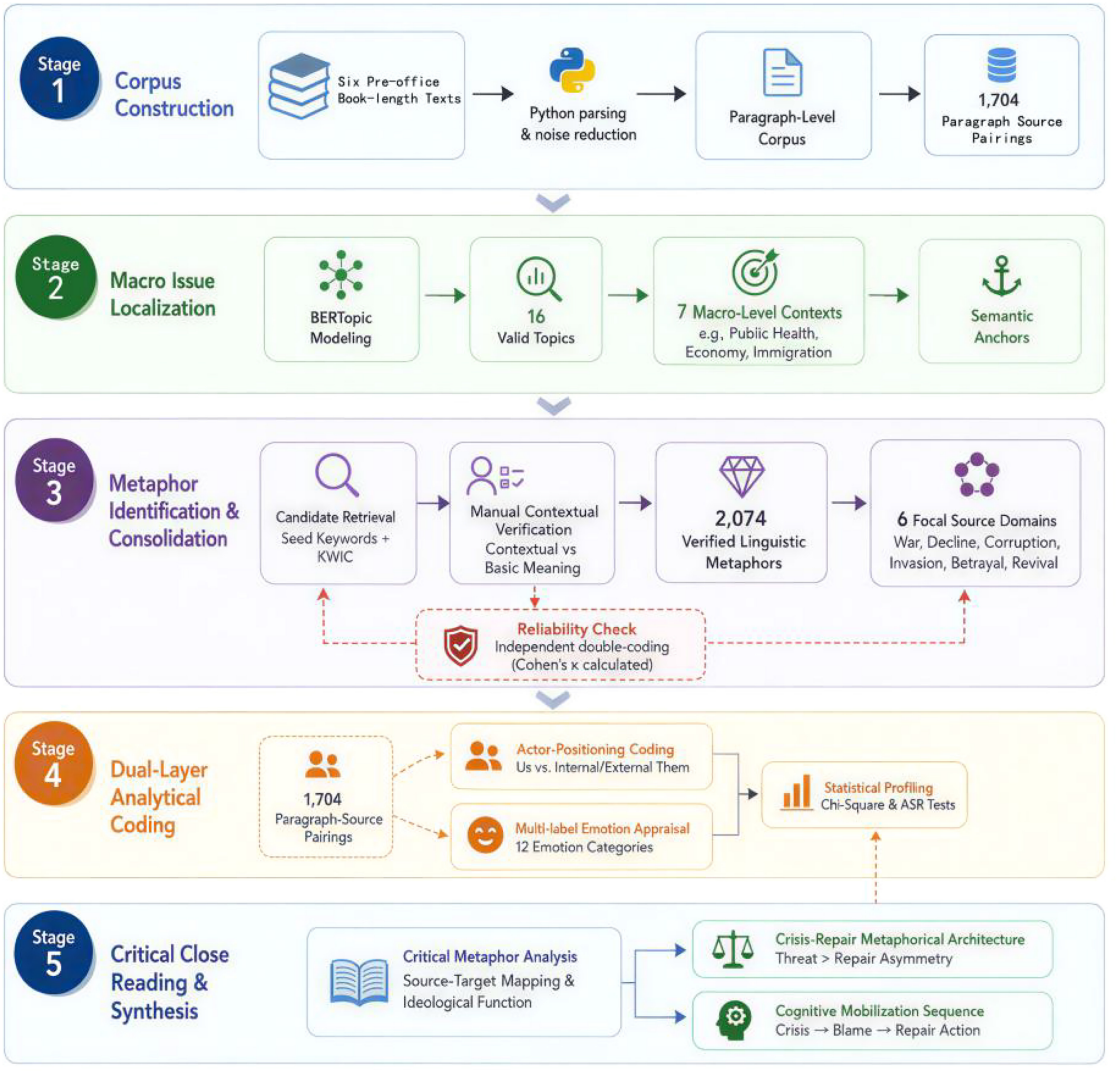
Corpus-assisted critical metaphor analysis workflow.

Figure 1 summarizes the overall analytical workflow.

To strengthen reliability control, a second trained coder independently coded a blinded stratified random sample of 720 candidate instances drawn from the full set of keyword occurrences. The reliability check focused on the two manual decisions most central to the metaphor analysis: metaphorical-status judgment and source-domain assignment. Agreement was 80.0% (Cohen’s κ = 0.556) for the binary metaphorical-status decision and 79.72% (Cohen’s κ = 0.749) for the seven-category classification combining the six focal source domains and excluded cases. Among the 401 instances that both coders independently identified as metaphorical, agreement on source-domain assignment was 99.50% (Cohen’s κ = 0.994). These results indicate that source-domain assignment was highly stable once metaphorical status had been established, whereas the main site of interpretive variation lay at the inclusion–exclusion boundary between metaphorical and non-metaphorical usage.

For actor-positioning coding, reliability was assessed separately because its coding unit and variables differ from keyword-level metaphor identification. A second trained coder independently coded a blinded stratified random sample of 315 metaphor-bearing paragraph–source-domain pairings. Agreement for the seven-category positioning configuration was 65.1% (Cohen’s κ = 0.557). A derived binary measure of out-group presence showed stronger agreement, 86.0% (Cohen’s κ = 0.681), and agreement for the internal/external distinction reached 96.2% (Cohen’s κ = 0.791). Open-ended fields, including in-group and out-group labels, were used to support interpretation and were resolved through consistency review rather than κ statistics. Emotion appraisal was not included in these reliability calculations because it was used as a secondary interpretive layer for comparing evaluative profiles across source domains, rather than as the primary basis for metaphor identification or actor-positioning classification.

## 4 Results

### 4.1 Issue heterogeneity as a test condition for shared discourse

The BERTopic results show that the corpus is thematically heterogeneous rather than organized around a single issue field, making it possible to test whether these texts nevertheless converge on a shared metaphorical repertoire. Using the parameter configuration described in section 3.2, the model identified 16 valid topics, while 914 paragraphs, accounting for 11.57%, were assigned to the outlier topic, topic-1. Table 4 reports the number of paragraphs, representative keywords, and manually assigned issue categories for each topic.

**TABLE 4:** BERTopic topic modeling results and manually assigned issue categories.

Topic	Number of paragraphs	Representative keywords	Manually assigned issue category
-1	914	OUTLIER	Outlier paragraphs, not included in the main analysis
0	3350	Fauci, AIDS, vaccine, Gates, COVID, health, NIAID, drug	Public health, vaccine politics, and medical authority
1	741	Education, Christian, schools, classical, progressive, students	Education, religious tradition, and culture war
2	516	FBI, DOJ, Russia, investigation, deep state, intelligence	Judicial institutions, investigative politics, and the deep state
3	499	Mamaw, mom, Papaw, family, Middletown, home	Family, local community, and white working-class experience
4	238	Defense, military, DoD, secretary, chairman	Defense system, military bureaucracy, and national security
5	226	Economy, financial, stock, corporate, workers, banks	Economic decline, financial capital, and working-class anxiety
6	225	Immigrants, border, immigration, party, America, Republican	Immigration, borders, and national identity boundaries
7	218	Free speech, democracy, elite, Google, censorship, vote	Free speech, democratic legitimacy, and elite control
8	206	Racism, Frankfurt School, critical race theory, white, Marxism	Race politics, critical theory, and the cultural Left
9	164	China, Chinese Communist Party, Beijing, Taiwan, trade	China’s rise, competition with China, and global order
10	131	Gender, transgender, women, puberty, children	Gender politics, child protection, and identity controversies
11	116	Religious, God, faith, constitution, liberty	Religious freedom, faith politics, and constitutional rights
12	107	Nuclear, war, Putin, Ukraine, Russia	Nuclear war, the Russia–Ukraine conflict, and anti-interventionist diplomacy
13	99	Marine, Iraq, soldiers, boot camp, military	Military experience, war experience, and national service
14	75	Yale, law school, Ohio State, job, class	Elite education, class mobility, and cultural rupture
15	73	Working-class, poor, jobs, work, people	White working class, poverty, and labor experience

Based on keywords, representative paragraphs, and dominant semantics, the 16 valid topics were aggregated into seven macro-level contexts, as shown in Table 5. These contexts range from public health, education, and political institutions to family and class mobility, global order, economic decline, and immigration. The corpus is therefore not organized around a single policy field. Precisely because the texts are thematically heterogeneous, any recurrent metaphorical patterns identified below provide stronger evidence of cross-issue integration rather than mere topical similarity.

**TABLE 5:** Seven macro-level contexts after issue aggregation.

Macro-level context	Corresponding topics
Public health, vaccine politics, and medical authority	0
Education, religious tradition, and culture war	1, 8, 10, 11
Political institutions, judicial institutions, and the deep state	2, 7
Family, local community, and class mobility	3, 14, 15
Global order, the defense system, and war risk	4, 9, 12, 13
Economic decline, financial capital, and working-class anxiety	5
Immigration, borders, and national identity boundaries	6

### 4.2 Crisis-Repair asymmetry in the overall metaphorical repertoire

The overall distribution of the six retained source domains reveals a clear crisis-repair asymmetry, with threat-oriented metaphors dominating the corpus and revival/reconstruction providing a more limited future-oriented script of repair. Table 6 summarizes the overall frequencies and proportions of the six retained focal source domains. The retrieval procedure generated 3,660 candidate keyword occurrences, of which 2,074 were confirmed as contextually verified linguistic metaphorical expressions after manual verification.

**TABLE 6:** Overall frequencies and proportions of the six retained focal source domains.

Source domain	Number of metaphorical instances (N)	Proportion (%)
War/conflict	976	47.1
Decline/fall	408	19.7
Corruption/disease	290	14.0
Revival/reconstruction	196	9.5
Invasion/infiltration	117	5.6
Betrayal/abandonment	87	4.2
Total	2,074	100.0

The six domains form a pronounced crisis-repair asymmetry. The five threat-oriented domains—war/conflict, decline/fall, corruption/disease, invasion/infiltration, and betrayal/abandonment—account for 1,878 instances (90.55%), whereas revival/reconstruction accounts for 196 instances (9.45%). War/conflict alone comprises 47.1%, followed by decline/fall (19.7%) and corruption/disease (14.0%). The corpus therefore devotes more metaphorical resources to naming threats, diagnosing damage, and assigning responsibility than to elaborating repair. Revival/reconstruction nevertheless provides the future-oriented endpoint through which crisis is translated into restoration and renewed collective agency.

### 4.3 Authorial signatures within a shared metaphorical repertoire

Authorial variation is visible in the corpus, but it operates as differences in metaphorical emphasis rather than as a fragmentation of the shared crisis-and-repair repertoire.

The association between author and source domain is statistically significant, χ^2^(25) = 328.106, *p* < 0.001, but modest in magnitude (Cramér’s *V* = 0.178), indicating that author identity modulates metaphorical emphasis without reorganizing the shared repertoire. The adjusted standardized residuals (ASR) reveal three broad profiles. Table 7 reports the frequency and percentage distribution of metaphorical source domains by author. The first is institutional and bodily pathology: Patel shows elevated corruption/disease (ASR = 8.909), and Kennedy shows elevated corruption/disease (ASR = 5.548) and invasion/infiltration (ASR = 5.422). The second is conflictual mobilization: Gabbard shows elevated war/conflict (ASR = 8.947), and Hegseth shows elevated war/conflict (ASR = 2.481). The third consists of decline-and-repair narratives: Rubio shows elevated decline/fall (ASR = 7.206), and Vance shows elevated betrayal/abandonment (ASR = 2.178) and revival/reconstruction (ASR = 2.399). All these residuals are significant at *p* < 0.05, with those for Patel, Gabbard, Kennedy, and Rubio significant at *p* < 0.01. These patterns represent author-specific configurations of a common crisis-and-repair grammar rather than separate metaphorical systems.

**TABLE 7:** Frequencies and percentages of metaphorical source domains by author.

Author	Statistic	War/ conflict	Invasion/ infiltration	Corruption/ disease	Decline/ fall	Betrayal/ abandonment	Revival/ reconstruction	Total
Kash Patel	N	164	9	97	24	9	24	327
	%	50.15	2.75	29.66	7.34	2.75	7.34	100
Tulsi Gabbard	N	207	6	9	33	17	18	290
	%	71.38	2.07	3.10	11.38	5.86	6.21	100
Robert F. Kennedy Jr.	N	266	68	143	150	30	67	724
	%	36.74	9.39	19.75	20.72	4.14	9.25	100
Pete Hegseth	N	134	9	16	57	5	25	246
	%	54.47	3.66	6.50	23.17	2.03	10.16	100
J. D. Vance	N	110	8	15	51	16	32	232
	%	47.41	3.45	6.47	21.98	6.90	13.79	100
Marco Rubio	N	95	17	10	93	10	30	255
	%	37.25	6.67	3.92	36.47	3.92	11.76	100

### 4.4 Contextual specialization and cross-domain extension of metaphors

The distribution of source domains across issue contexts reveals both contextual specialization and cross-domain extension, indicating that similar metaphorical resources are adapted to different policy fields rather than confined to a single issue domain. Table 8 reports the corresponding distributions across the seven macro-level contexts.

**TABLE 8:** Percentage distribution of metaphorical source domains across seven macro-level issue contexts, row percentages^1^.

Macro-level context	War/conflict	Invasion/ infiltration	Corruption/ disease	Decline/fall	Betrayal/ abandonment	Revival/ reconstruction	Total
Public health, vaccine politics, and medical authority	36.57	9.28	19.81	20.78	4.29	9.28	100
Education, religious tradition, and culture war	57.00	3.41	5.46	21.50	3.41	9.22	100
Political institutions, judicial institutions, and the deep state	51.24	2.17	26.71	7.76	3.73	8.39	100
Family, local community, and class mobility	44.59	3.15	6.76	25.23	7.21	13.06	100
Global order, the defense system, and war risk	64.19	4.96	5.51	15.43	3.86	6.06	100
Economic decline, financial capital, and working-class anxiety	12.35	0.00	7.41	60.49	3.70	16.05	100
Immigration, borders, and national identity boundaries	44.32	9.09	5.68	25.00	2.27	13.64	100

^1^The statistical unit in this table is “metaphor–context assignment.” Since Topic 15, “white working class, poverty, and labor experience,” simultaneously involves local community, class mobility, and economic labor experience, it is assigned to both “family, local community, and class mobility” and “economic decline, financial capital, and working-class anxiety.” Therefore, the total N in this table is 2,091, slightly higher than the total number of metaphorical instances, 2,074.

The association between source domain and issue context is statistically significant, χ^2^(30) = 326.52, *p* < 0.001, but modest in magnitude (Cramér’s *V* = 0.177), indicating contextual specialization without strict topic confinement. Three contrasts are revealing. War/conflict is most prominent in global order and war risk (64.19%) but also extends into education and culture war (57.00%), showing the domestic expansion of a conflict frame. Decline/fall reaches 60.49% in the economic context, organizing economic change as historical downward movement. Corruption/disease links political institutions (26.71%) with public health (19.81%) through a shared pathology frame, while invasion/infiltration connects public health (9.28%) and immigration (9.09%) through analogous bodily and territorial boundary scripts. The pattern therefore combines contextual specialization with cross-domain transfer.

### 4.5 Actor positioning in metaphorical boundary-making

The actor-positioning analysis shows that the six focal source domains do not merely name crises; they also allocate political roles within recurrent in-group/out-group configurations. Table 9 reports the distribution of actor-positioning configurations by metaphorical source domain. Across 1,704 metaphor-bearing paragraph–source-domain pairings, 1,404 pairings, or 82.4%, contained explicit or contextually recoverable collective positioning. The most frequent configuration was labeled “us vs. internal them” (*N* = 541, 31.7%), followed by “internal them only” (*N* = 432, 25.4%). By contrast, externalized out-group configurations were much less frequent: “us vs. external them” accounted for 78 pairings (4.6%), and “external them only” accounted for 22 pairings (1.3%).

**TABLE 9:** Distribution of actor-positioning configurations by metaphorical source domain.

Source domain	No collective positioning	In-group only	External them only	Internal them only	Us vs. external them	Us vs. internal them	Mixed/ambiguous	Total
War/conflict	109	73	7	170	38	255	74	726
Decline/fall	75	32	7	78	21	95	42	350
Corruption/disease	41	10	1	88	2	78	32	252
Revival/reconstruction	37	26	1	29	5	66	13	177
Invasion/infiltration	22	9	4	44	5	23	8	115
Betrayal/abandonment	16	5	2	23	7	24	7	84
Total	300	155	22	432	78	541	176	1,704

The association between metaphorical source domain and actor-positioning configuration was statistically significant, χ^2^(30) = 87.882, *p* < 0.001, Cramér’s *V* = 0.102. Although the effect size is modest, the result suggests that source domains differ in both semantic scripts and how they position collective political actors. Overall, the corpus constructs political crisis less as a simple opposition between the nation and external enemies than as a recurrent struggle between an imagined political “us” and internal political, institutional, or ideological opponents.

### 4.6 Diagnostic emotion-appraisal profiles across source domains

The emotion-appraisal results reveal a distinction between a shared affective background and domain-diagnostic evaluative profiles. Based on 1,704 paragraph–source-domain pairings, fear/anxiety occurred in 95.54% of the cases overall and should therefore be interpreted as a general crisis background rather than as a distinguishing feature of any single source domain. The more informative contrasts concern emotions that were significantly over- or underrepresented across source domains. Except for determination, all emotion labels were significantly associated with source-domain type, *p* < 0.001, with hope showing the largest difference, χ^2^(5) = 76.27, Cramér’s *V* = 0.212. Table 10 therefore summarizes diagnostic emotion-appraisal profiles rather than general emotional co-occurrence.

**TABLE 10:** Diagnostic emotion-appraisal profiles across metaphorical source domains.

Metaphorical source domain	Significantly overrepresented (ASR)	Significantly underrepresented (ASR)
War/conflict	Anger 2.06; pride/dignity 2.08	Loss/nostalgia −3.79
Invasion/infiltration	No significant positive overrepresentation	Loss/nostalgia −3.59; pride/dignity −2.59
Corruption/disease	Disgust/pollution 5.74; humiliation 4.95; anger 3.19; fear/anxiety 2.06	Hope −2.98; pride/dignity −3.50; sacredness −4.78
Decline/fall	Loss/nostalgia 5.64; sacredness 2.31	Anger −3.14; fear/anxiety −3.02; disgust/pollution −2.68
Betrayal/abandonment	Humiliation 3.80; disgust/pollution 3.14; betrayal 2.89; anger 2.63	Hope −3.80; collective efficacy −2.92; sacredness −2.53
Revival/reconstruction	Hope 7.62; sacredness 4.26; pride/dignity 3.84; justice appeal 2.38	Anger −3.79; disgust/pollution −2.81; humiliation −2.77

Overall, these results suggest that the emotional contribution of metaphor lies less in simple one-to-one associations than in the contrast between a shared crisis background and domain-specific evaluative profiles. Threat-oriented domains differentiate crisis into confrontation, boundary violation, internal pathology, historical decline, and moral betrayal. Revival/reconstruction, by contrast, is marked by a concentrated repair-oriented profile, especially through hope, sacredness, pride/dignity, and justice appeal. This contrast helps explain how the corpus moves from crisis construction and blame attribution toward a more focused promise of repair and collective renewal.

## 5 Discussion

### 5.1 Cognitive mappings of metaphor

The distributional analysis in section 4 captures recurrent linguistic realizations of metaphor. Frequencies and distributions chart the overall configuration of metaphorical resources. To understand how these metaphors construct political meaning, however, it is necessary to return to specific contexts and examine how experiential structures from source domains are projected onto political target domains. As described in section 2.1, the six retained focal source domains invoke distinct experiential scripts—friend-enemy confrontation, historical downward movement, internal pathology, boundary defense, moral breach, and repair and redemption—which are examined below in specific political contexts. With this in mind, this section analyzes how the six metaphor types cast target domains such as U.S. politics, education, immigration, the economy, public health, and global competition into crisis narratives that are perceptible, evaluative, and action-oriented.

#### 5.1.1 War/conflict metaphors: cross-domain extension of a confrontational frame

As the most frequent source domain in the corpus, war/conflict metaphors extend a confrontational frame across multiple issue domains. Hegseth’s *Battle for the American Mind* provides a representative example of the cross-domain extension of this metaphor. The text conceptualizes the time children spend in

K–12 schooling as a “16,000-hour war,” maps the classroom onto a “battlefield” for children’s minds, describes progressives as occupying the “commanding heights,” and portrays conservative parents as facing “fortified machine-gun nests” (Hegseth and Goodwin, 2022). Within this frame, education is represented as an existential struggle over children’s souls, the nation’s future, and the continuity of civilization, rather than as a matter of curriculum design, pedagogical philosophy, or public policy debate.

A similar logic is extended to the judicial and public health domains. Patel presents law as something that can be “weaponize[d]” (Patel, 2023), while Kennedy incorporates COVID-19 and its governance into the narrative structure of a “war against COVID” (Kennedy, 2021). War/conflict metaphors thus form a transferable grammar of confrontation. They transform non-military issues such as education, law, public health, and identity politics into relations among battlefields, enemies, positions, and counterattacks, thereby lending political disagreement a stronger sense of urgency and a more zero-sum character. The ideological function of this metaphor lies in its reinforcement of friend–enemy division and the logic of victory and defeat, while narrowing the imagined space for negotiation, compromise, and procedural solutions.

#### 5.1.2 Decline/fall metaphors: a downward historical narrative

Decline/fall metaphors provide a vertical temporal structure for crisis narratives. Rubio’s *Decades of Decadence* is a representative example. The text summarizes changes in the United States over recent decades through expressions such as “internal collapse,” “decadent choices,” and “America’s decline,” organizing transformations in economic structure, family stability, community life, and national identity into an overarching downward trajectory. Even after mutually exclusive issue classification, decline/fall metaphors remain the most frequent in the economic context, indicating that economic issues are narrated through frames of historical descent, industrial loss, and communal breakdown (Rubio, 2023).

Within this metaphorical structure, manufacturing is more than an economic sector. It is endowed with moral significance as a foundation for family life, community stability, and national strength. Its “decline,” therefore, is more than a reduction in industrial competitiveness: it is described as the disintegration of working-class life and local community structures (Rubio, 2023). Vance similarly materializes the decline of Middletown through images such as “broken windows” and abandoned stores, turning class descent into a visible social experience (Vance, 2016). The function of decline/fall metaphors is to transform complex economic and social changes into a historical contrast between a once-whole past and a now-broken present, thereby providing the emotional precondition for narratives of revival.

#### 5.1.3 Corruption/disease metaphors: the organismic politics of internal pathology

Corruption/disease metaphors construct institutions, organizations, or social relations as bodies damaged, decayed, or contaminated from within. Patel’s *Government Gangsters* presents a typical narrative of institutional decay. The federal bureaucracy is described as “rotten at the top,” while corruption is constructed as a spreading internal pathology, as in the phrase “rot has spread and manifested itself” (Patel, 2023). In this mapping, institutional problems are no longer merely failures of procedure or checks and balances. They are understood as the diffusion of lesions inside a body politic.

Kennedy’s text, by contrast, forms a “double contamination” narrative that juxtaposes bodily contamination with institutional corruption. On the one hand, drugs, vaccines, and viral transmission are situated within a bodily risk frame. On the other hand, relations among public health agencies, medical research, and pharmaceutical capital are portrayed as a process through which institutional space is eroded (Kennedy, 2021). The key effect of corruption/disease metaphors is to transform political judgment into a logic of diagnosis, removal, and restoration of health. This strengthens the cognitive legitimacy of forceful intervention, while tending to suppress attention to the complexities of professional disagreement, evidentiary uncertainty, and procedural coordination in institutional governance.

#### 5.1.4 Invasion/infiltration metaphors: boundary anxiety and fear of contamination

Although less frequent overall, invasion/infiltration metaphors are concentrated in two domains—immigration/borders and public health—suggesting that boundary anxiety travels across territorial and bodily registers

Rubio’s discussion of the border presents a fluid narrative organized around boundary violation. The border is mapped onto “floodgates,” while migration is described through expressions such as “flood,” “surge,” and “poured in.” National resources and public order are correspondingly portrayed as defensive lines at risk of being “overwhelming” (Rubio, 2023). Within this form of expression, individual migrants are reconstructed as an undifferentiated inflow that exceeds the state’s capacity to respond. Gabbard further links border opening with urban disorder. Through expressions such as “opening our borders to millions of illegal, unvetted migrants” and “our cities to be overrun with crime,” she compresses the immigration issue into a one-directional causal chain of boundary failure, external inflow, and internal disorder (Gabbard, 2024). In the public health context, Kennedy compares viral transmission to an invading force gaining a “beachhead” and to an unstoppable “tide,” thereby constructing both bodily boundaries and the population community as spaces vulnerable to occupation (Kennedy, 2021).

#### 5.1.5 Betrayal/abandonment metaphors: moral accusation against the internal other

Betrayal/abandonment metaphors are the least frequent source domain but carry concentrated moral intensity. This metaphor centers on the rupture of trust: crisis is explained as an active abandonment of the people, the nation, the Constitution, or one’s class origins by members within the community.

Vance’s text provides a distinctive form of this metaphor within class experience. Expressions such as “abandoned son” and “class betrayal” place paternal absence, family rupture, and the psychological conflict of class mobility within a moral frame of abandonment and estrangement from one’s origins. Upward mobility therefore has a double meaning: it signifies personal success, but it may also imply an emotional break from the original community (Vance, 2016). Gabbard directly politicizes this logic through the expression “The Democrat elite have betrayed us all,” organizing anti-establishment sentiment as political awakening after deception and betrayal (Gabbard, 2024). Patel further anchors betrayal in the constitutional oath, portraying violations of public duty as moral rupture and using this logic to separate national security elites from the political community (Patel, 2023).

The function of betrayal/abandonment metaphors is to transform political contradiction into moral breach and to channel complex institutional conflicts toward identifiable internal others. Compared with the external enemy constructed by war metaphors, betrayal metaphors create an “enemy within.” Their emotional intensity is therefore closer to anger, humiliation, and punitive justice.

#### 5.1.6 Revival/reconstruction metaphors: scarce redemption and the call to action

Revival/reconstruction metaphors are the most future-oriented of the six retained source domains. Rubio’s text illustrates this logic at the national level. He calls for efforts to “restore common-good capitalism” and “rebuild our national strength,” transforming economic nationalism into a mission of national reconstruction (Rubio, 2023). Patel, meanwhile, emphasizes institutional repair, framing reformers as actors who restore institutional trust by enforcing accountability (Patel, 2023). Vance presents a more micro-level form of revival at the level of family and locality: “Dad’s life project was rebuilding for himself what he once had in Kentucky.” Here, the restoration of individual life order is understood as a reconstruction of earlier communal experience (Vance, 2016).

The role of revival/reconstruction metaphors is to provide a future-oriented outlet for the crisis, loss, and anger accumulated by the preceding threat-oriented metaphors. War, invasion, corruption, decline, and betrayal jointly construct crisis, while revival/reconstruction directs crisis toward repair, taking back, and beginning again. It allows political action in these texts to be understood as defense and punishment, while also serving as a promise directed toward the future.

### 5.2 Diagnostic emotion-appraisal profiles and the cognitive mobilization sequence

The cognitive mobilization process outlined in section 2.3 takes the specific form of a cognitive mobilization sequence in these texts. It describes a textual argumentative architecture, not a chronological progression in readers’ minds: threat-oriented and repair-oriented source domains jointly form an evaluative structure in which crisis construction, blame attribution, and repair imagination become mutually reinforcing.

Fear/anxiety forms a shared crisis background across the corpus, while other emotions differentiate the direction of crisis interpretation. Anger and pride/dignity reinforce confrontational resistance; disgust/pollution and humiliation support the pathologizing of institutions or elites; loss/nostalgia gives decline/fall metaphors a temporal structure of historical damage; and betrayal, humiliation, and anger moralize crisis as internal breach. These differentiated profiles specify how metaphorical source domains assign responsibility, intensify boundary-making, and make particular responses appear necessary.

This pattern also clarifies the crisis–repair asymmetry of the corpus. The five threat-oriented domains—war/conflict, invasion/infiltration, corruption/disease, decline/fall, and betrayal/abandonment—elaborate the naming of threat, diagnosis of damage, and attribution of blame. Revival/reconstruction is much less frequent, but it is emotionally more specialized: its association with hope, sacredness, pride/dignity, and justice appeal provides a repair-oriented outlet for the crisis emotions accumulated by the other domains. In this sense, the emotional contribution of metaphor lies in a movement from shared crisis affect to differentiated blame and finally to a concentrated promise of restoration and collective renewal.

The significance of these action scripts lies in their cumulative ordering. The threat-oriented domains do not simply multiply negative images; they distribute crisis into different problem types—conflict, boundary breach, pathology, decline, and moral breach. Each problem type assigns a different kind of antagonist and makes a different response appear appropriate. Revival/reconstruction then condenses these dispersed crisis appraisals into a future-oriented promise of repair. The cognitive mobilization sequence is therefore not a chronological process in readers’ minds, but a textual architecture in which metaphorical framing, actor positioning, and emotion appraisal jointly move the discourse from threat recognition to blame attribution and finally to repair imagination.

The actor-positioning analysis further specifies this cognitive mobilization sequence. The corpus locates “them” within the political community more often than it constructs crisis as a conflict between the nation and an external enemy: elites, bureaucratic actors, institutional authorities, and ideological opponents are framed as corrupting, betraying, or obstructing an imagined “us.” External actors, including migrants, foreign competitors, and hostile states, remain important, particularly in invasion/infiltration and war/conflict frames, but they are less central to the overall configuration. Metaphors therefore organize collective roles as well as crisis meanings: “us” is constructed as threatened, morally entitled to resist, and capable of restoration, while “them” becomes the target of defense, exposure, punishment, or exclusion.

### 5.3 Theoretical contributions

The findings show that research on Trumpism can move beyond campaign rhetoric and leader-centered communication. The six pre-office texts examined here use a shared crisis-and-repair metaphorical structure across different issue domains. This shared structure reflects cognitive and narrative resources available to governing-coalition elites. This study contributes to Trumpism studies by treating Trumpist discourse as a broader textual formation. Through this formation, governing-coalition elites construct crisis, identify antagonists, and imagine repair. Future research can examine whether this architecture also appears in post-office texts and in the discourse of elites outside the governing coalition.

A second theoretical contribution is to clarify how metaphor, emotion appraisal, and actor positioning work together to organize political cognition at the textual level. The analysis shows that the retained source domains provide more than isolated rhetorical images. They organize crisis into different problem types, including conflict, boundary breach, pathology, decline, and moral betrayal. Each problem type assigns a particular antagonist and points toward a particular response logic. This finding refines critical metaphor analysis by explaining how metaphorical framing, emotional evaluation, and collective role allocation reinforce one another in the construction of political meaning.

Conceptually, the differentiated emotion-appraisal profiles show that a single source-domain family can frame a political problem, evaluate it emotionally, and suggest a course of action at the same time. The “cognitive mobilization sequence” described in section 5.2 is best understood as a discursive architecture: a textual pattern in which crisis construction, blame attribution, and repair imagination reinforce one another. It should not be treated as a psychological mechanism or as evidence of audience effects.

### 5.4 Methodological contributions

Methodologically, the study demonstrates how BERTopic-based issue localization can be integrated with corpus-assisted metaphor identification, source-domain mapping, actor-positioning coding, and multi-label emotion appraisal in a single analytical workflow. In this workflow, computational outputs are treated as semantic anchors for critical close reading rather than as self-sufficient evidence. This design is consistent with the study’s use of topic modeling for macro-level issue localization rather than for directly determining metaphor interpretation.

The reliability results further indicate that the main interpretive friction lies at the inclusion–exclusion boundary of metaphorical status rather than in source-domain assignment. This finding has methodological implications for future corpus-assisted metaphor studies: coding protocols should devote particular attention to distinguishing literal, metaphorical, and contextually ambiguous uses of source-domain vocabulary before source-domain classification is conducted.

### 5.5 Limitations and future directions

This study has several limitations. First, the corpus is limited to six pre-office books, and the conclusions cannot be directly generalized to post-office policy speeches, campaign speeches, social media texts, or the broader field of right-wing discourse. Future research may include different time points and text genres to examine the evolution of metaphorical frames.

A related limitation concerns the potential affinity between political genre, public prominence, and conflict narration. Although the corpus was selected according to pre-office authorship and public visibility, highly visible political elite books may themselves be predisposed toward crisis and conflict narration, and may gain public circulation partly through such dramatization. The findings should therefore be understood as identifying a recurrent crisis-and-repair metaphorical architecture within prominent Trumpist governing-coalition texts, rather than as establishing the baseline frequency of these metaphors in political discourse as a whole. Future research could test the generalizability of this architecture by incorporating non-political bestselling nonfiction, ideologically diverse political corpora, or a more systematic random or stratified sampling frame.

Second, the six texts differ in length and issue concentration. In particular, Kennedy’s text is considerably longer, which may affect the weight of public health topics and the overall frequency of metaphorical instances. Future studies may adopt measures such as metaphor density per ten thousand words, author-level normalized statistics, or sensitivity tests excluding a single long text.

Third, metaphor identification and critical interpretation involve contextual judgment, especially at the boundary between metaphorical and non-metaphorical usage. Although the present study incorporated a stratified intercoder reliability check, agreement was stronger for source-domain assignment than for the initial inclusion–exclusion decision. The six focal source domains should therefore be understood as selective analytical categories derived from recurrent and theoretically relevant patterns in the corpus, rather than as a complete taxonomy of all metaphorical expressions. Future research may extend double coding to the full corpus, compare multiple coding teams, or test the transferability of the coding scheme in other political corpora.

Finally, emotion appraisal in this study is used as a secondary interpretive layer for comparing evaluative profiles, rather than as evidence of authors’ intentions, actual audience cognition, or behavioral effects. Future studies may combine audience reception research, experimental design, or communication-effect analysis for further validation. The study therefore reconstructs discourse-level conceptual patterns from textual evidence, without claiming to directly observe authors’ or readers’ mental representations.

## 6 Conclusion

This study examines six pre-office books written by political figures who later entered core positions in the Trump governing coalition. Using a corpus-assisted critical metaphor analysis, it examines the distributional structure, cognitive mappings, and diagnostic emotion-appraisal profiles associated with metaphorical resources in these texts. The study identifies a total of 2,074 contextually verified linguistic metaphorical expressions across the six texts, concentrated in six retained focal source domains: war/conflict, decline/fall, corruption/disease, invasion/infiltration, betrayal/abandonment, and revival/reconstruction. There is a significant but weak association between author identity and metaphorical source domain, Cramér’s V = 0.178, indicating that although the six authors differ in issue emphasis and expressive style, they draw on a relatively stable repertoire of crisis-and-repair-oriented metaphorical resources.

The analysis reveals a recurrent crisis-and-repair architecture across six heterogeneous pre-office texts. Five threat-oriented domains—war/conflict, decline/fall, corruption/disease, invasion/infiltration, and betrayal/abandonment—together elaborate threat naming, damage diagnosis, and blame attribution, while revival/reconstruction provides a concentrated future-oriented outlet. Fear/anxiety supplies the shared affective background; domain-specific emotions—anger and pride/dignity for war/conflict, loss/nostalgia for decline/fall, disgust/pollution and humiliation for corruption/disease, hope and sacredness for revival/reconstruction—differentiate the direction of crisis interpretation. Actor positioning reinforces this structure: the predominant ‘us vs. internal them’ configuration (31.7%) constructs primary antagonists inside the political community—as corrupting elites, betraying insiders, or obstructing institutional actors—rather than as external enemies.

These profiles clarify how metaphorical framing, actor positioning, and emotional appraisal jointly form discursively constructed action scripts, rather than providing evidence of actual audience behavior or measured political effects.

This study remains limited by corpus scope, the potential affinity between prominent political elite writing and conflict narration, differences in text length, the interpretive boundary between metaphorical and non-metaphorical usage, and the secondary interpretive role of emotion appraisal. Nevertheless, its analysis helps explain how the Trumpist governing coalition constructs a shared political reality at the textual level.

## Data Availability

The source texts analyzed in this study are the six published book-length works listed in Table 1 and are subject to copyright restrictions. The authors therefore cannot redistribute the full texts. Non-copyrighted derived data supporting the conclusions of this article, including coding labels and statistical outputs, will be made available by the corresponding author JL, lvjiaxuan@nudt.edu.cn, upon reasonable request.
